# Re-Analysis of the Uncertainty of the 0.895 µm Diameter (NIST SRM^®^ 1690) and the 0.269 µm Diameter (NIST SRM^®^ 1691) Sphere Standards

**DOI:** 10.6028/jres.110.004

**Published:** 2005-02-01

**Authors:** G. W. Mulholland

**Affiliations:** National Institute of Standards and Technology, Gaithersburg, MD 20899

**Keywords:** light scattering, Mie Scattering, polystyrene spheres, Standard Reference Materials, transmission electron microscopy, uncertainty analysis

## Abstract

The uncertainties of the mean diameters of the nominal 1.0 µm SRM^®^ 1690 polystyrene spheres and of the nominal 0.3 µm SRM^®^ 1691 polystyrene spheres are recomputed using the current NIST Guidelines for computing uncertainty. The revised expanded uncertainty (approximately 95 % confidence level) for SRM^®^ 1690 polystyrene spheres is equal to 0.005 µm compared to previous value of 0.008 µm. The revised expanded uncertainty for SRM^®^ 1691 is equal to 0.004 µm compared to the previous value of 0.007 µm. The major cause of the reduction in the uncertainty for the 1.0 µm spheres is from a decrease in the recomputed uncertainty of the refractive index of the polystyrene spheres. The 1.0 µm spheres were used in calibrating the electron microscope used to size the 0.3 µm spheres, and the reduction in the uncertainty of 1.0 µm SRM^®^ uncertainty was the biggest factor in the decrease in the uncertainty of the 0.3 µm spheres.

## 1. Introduction

The NIST Standard Reference Material 1690 (NIST SRM^®^ 1690) consists of a nearly monosize suspension of 0.895 µm polystyrene spheres in water at a mass fraction of approximately 0.5 %. This standard is used in the certification of secondary standards and also used directly in the calibration of electron microscopes, of scanning surface inspection systems in the semiconductor industry, and of other particle sizing instruments when the most accurate sizing standards are needed.

The certification of SRM^®^ 1690 was based on the measurement of light scattering intensity versus scattering angle for a diluted suspension of the polystyrene spheres. Key features of the experiment [1] were the use of an intensity stabilized laser, an accurately indexed rotary table, and photon counting detection. The particle diameter was determined from a nonlinear least squares fit of the predicted scattering based on Mie theory and the measured data.

The uncertainty analyses used in this study [1] is not consistent with the current NIST policy [2,3] for reporting measurement uncertainty and result in overestimates of those uncertainties. The focus of this note is the recalculation of the uncertainty per the 1994 Guidelines [2]. There is also an updated analysis of the refractive index component of the uncertainty.

NIST SRM^®^ 1690 was also used in the measurement of SRM^®^ 1691 (0.269 µm diameter polystyrene spheres). The effect of the change in the uncertainty in SRM^®^ 1690 on the uncertainty for SRM^®^ 1691 is determined. The uncertainty in the SRM^®^ 1691 is also recomputed per the 1994 guidelines, since the methodology used previously [4] is not consistent with current NIST policy.

## 2. Calculation of Expanded Uncertainty

The old procedure for computing the uncertainty is briefly reviewed and then the revised uncertainty analysis is presented based upon the 1994 Guidelines.

### 2.1 Old Method

The total uncertainty, *U*_T(old)_, was computed by adding the random error, *R*, and the sum of the absolute values of the systematic errors, *u*_B_*_i_*^1^.

$$U_{T(\text{old})}=R+\sum_{i}\left|u_{Bi}\right|$$ (1)

The random component of the uncertainty, *R*, was computed as the product of a coverage factor, *k*, for a 95 % confidence level times the uncertainty of the mean for 10 repeat measurements of the mean, *u*_r_.

$$u_{r}={\left[\frac{1}{n(n-1)}\sum_{i=1}^{n}{\left(D_{n,i}-{\bar{D}}_{n}\right)}^{2}\right]}^{1/2}.$$ (2)

The quantity *D_n,i_* is the average diameter of the *i*-th sample, and $\bar{D}$ is the average of the 10 samples. The computed value of *u*_r_ equals 0.000229 µm. The coverage factor *k* for 9 degrees of freedom based on Student’s t-distribution for “about 95 %” confidence interval is 2.32. Thus the value of *R* is given by the following:

$$R=ku_{r}=(2.32)(0.000229)=0.00053\;\text{ì m}.$$ (3)

The systematic uncertainties are related to the particle properties and the optical system. The uncertainties are expressed in terms of the effect on the particle diameter and the values are given in Table 1. The particle related uncertainties include the refractive index of the spheres, *u*_B1_, the presence of about 1 % agglomerated doublets, *u*_B2_, and multiple scattering from the particle suspension, *u*_B3_. The optical system related uncertainties include the reflection from the glass cell, *u*_B4_, the finite acceptance angle of the detector of about ± 1°, *u*_B5_, and the slight optical misalignment at zero angle, *u*_B6_. These uncertainties, which were referred to as systematic uncertainties in Mulholland et al. [1], are now classified as Type B uncertainties. These estimates are based on scientific judgment rather than based on statistical methods, as is done for Type A uncertainties.

The total uncertainty, *U*T(old) = 0.0074, is computed from Eqs. (1), (3), and the sum of the systematic uncertainties (see Table 1).

### 2.2 New Method

In 1994 the method for reporting uncertainties at NIST was unified [2] and aligned with the ISO Guide to the Expression of Uncertainty [3]. In this approach each component of uncertainty of a measurement result is represented by an estimated standard deviation, termed standard uncertainty with symbol *u*. There are two types of standard uncertainty. The first is computed by statistical means such as the standard deviation of the mean of several repeat measurements and is termed a Type A standard uncertainty. The second is often based on scientific judgment using all the relevant information available and is termed Type B standard uncertainty.

In the case of NIST SRM^®^ 1690, the Type A standard uncertainty is the standard deviation of the mean of 10 repeat measurements of the number mean diameter, which is defined as *u*_r_ in Eq. (2) and found to have a value of 0.000229 µm.

The Type B standard uncertainties for NIST SRM^®^ 1690 consist of six components of uncertainty given in Table 1 [1].

Following the NIST Guidelines, the combined uncertainty, *u*_c_, is computed as the root-sum-of-squares of the Type A uncertainties and the Type B uncertainties. The basis of this approximation is that provided the variables are independent, the variance of a sum of independent variables is equal to the sum of the variances.

$$u_{c}={\left[u_{r}^{2}+\sum_{i}u_{Bi}^{2}\right]}^{1/2}=0.0035.$$ (4)

The expanded uncertainty, *U*, which defines an interval having a level of confidence of about 95 % (95.4 %), is computed as *U* = *ku*_c_. The quantity *k* is the coverage factor and its value is dependent on the number of degrees of freedom for *u*_c_. In the limit of infinite degrees of freedom, the value of *k* is 2.0. For a finite number of degrees of freedom, *k* is estimated as the t-factor from the Student’s t-distribution based on the number of degrees of freedom and about a 95 % confidence interval. For a combined uncertainty arising from several components each with degrees of freedom *v_i_*, the effective number of degrees of freedom, *ν*_eff_, is estimated using the Welch-Satterthwaite formula [2]:

$$v_{\text{eff}}=\frac{u_{c}^{4}}{\sum_{i}\frac{c_{i}^{4}u_{i}^{4}}{v_{i}}}.$$ (5)

It is assumed that the number of degrees of freedom for each of the Type B terms in Eqs. (4) is infinity so that the only term in the sum is the Type A uncertainty given by Eqs. (2). In this case the sensitivity factor *c_i_* is unity, the term *u_i_* = *u_r_* = 0.000229 µm, and the degrees of freedom, *v_i_*, is 9 (the number of repeat measurements minus one). The resulting value of *ν*_eff_ from Eq. (5) is 4.8 × 10^5^. The corresponding value for *k* = 2.00. Given *k*, the value of the expanded uncertainty, *U*, is computed as 0.0070 µm.

### 2.3 New Method With Revised Type B Uncertainty Estimates

Several of the systematic uncertainties estimated by Mulholland et al. [1] are over estimates. The term “at most” is used in describing both the uncertainty associated with the reflections from the glass cell, *u*_B4_, and the finite acceptance angle of the detector, *u*_B5_. To convert these estimates to Type B standard uncertainties, we treat each of these quantities as having equal probability over the respective ranges of ± 0.001 µm for *u*_B4_ and ± 0.005 µm for *u*_B5_. For this rectangular probability distribution, the standard deviation is *u*_B_*_i_*/(3)^1/2^. So both of these uncertainties are reduced to 0.58 times their previous values, which corresponds to 0.0006 µm for *u*_B4_ and 0.0003 µm for *u*_B5_ (See corrected values in Table 1.)

The previous estimate of refractive index uncertainty corresponded to the range in the reported values from five studies [1]. This provides an over-estimate and the estimate could be revised by the method used above for *u*_B4_ and *u*_B5_. Instead, we compute the uncertainty based on the single particle refractive index measurements by Marx and Mulholland [5] for the SRM^®^ 1690 particles. This is a more accurate approach because two of the other four studies involved particles at least a factor of three smaller than the SRM^®^ and the other two studies, which used a method similar to [5], did not include a quantitative uncertainty analysis. The measurement of the refractive index was based on measuring the light scattering versus angle from 30° to 160° for a single, levitated SRM^®^ sphere. The refractive index and particle size were determined from best fits of Mie theory predictions to the scattering data for the incident laser polarization direction both parallel to the scattering plane and perpendicular to the scattering plane [5]. The best fit was based on the maximum in the harmonic mean, 1/*Q*_1_ + 1/*Q*_2_, of the results for the two polarization directions. The quantities *Q*_1_ and *Q*_2_ are the sums-of-squares of the differences between the measured and predicted scattering for the laser polarization direction parallel and perpendicular to the scattering plane. The resulting mean and standard deviation of the mean for measurements on eight separate particles is 1.6121 ± 0.0013.

There are two sources of Type B uncertainty for the refractive index: uncertainty in the angle, *u_θ_*, and in the polarization direction, *u*_P_. There was a slight drift in the encoder angle readout of 0.08° over the time that the measurements were made. Including this drift in the numerical simulation of the light scattering, it was found that the change in the refractive index was 0.005. We use this value as our estimate of *u_θ_*. It was found that for each polarization direction selected, there was about 0.5 % of that intensity of light with the orthogonal polarization direction. From numerical simulations, this effect was found to change the refractive index by 0.0032 (0.2 %). This is our estimate of *u*_P_.

The combined uncertainty in the refractive index, *u_n_*, is obtained as the quadrature sum of the standard deviation of the mean and the two Type B uncertainties. The resulting value is 0.0061. The effect of this refractive index uncertainty on the uncertainty in the diameter of SRM^®^ 1690, *u*_B1_, is determined to be 0.0020 µm based on the analysis on page 14 of the study by Mulholland et al. [1].

The combined uncertainty for the mean diameter is computed using Eq. (4) with the corrected values of *u*_B1_, *u*_B4_, and *u*_B5_. The resulting value is 0.0026 µm. Equation (5) is used for computing the degrees of freedom with a resulting value of 1.47 × 10^5^. For a 95 % confidence level, the corresponding coverage factor is 2.00. Therefore the corrected expanded uncertainty is 0.0052 µm. This value is about 1/3 less than the value of 0.008 µm on the SRM^®^ 1690 certificate.

## 3. Impact on the Certified Values for SRM^®^ 1691

The uncertainty of the 0.3 µm SRM^®^ is recomputed to include the effect of the change in the uncertainty in the 1.0 µm SRM^®^. The current NIST Guidelines for expressing uncertainty [2] are used in carrying out the analysis. The particle sizes for the 0.3 µm spheres were measured by transmission electron microscopy (TEM). Both the 1.0 µm SRM^®^ particles and the 0.3 µm particles were deposited on five TEM grids. For each grid, at least 40 of both the 0.3 µm particles and of the 1.0 µm particles were sized. The 1.0 µm SRM^®^ served as the magnification standard for the measurements. For each of the five TEM grids a mean size was computed. The average of these five mean sizes was found to be 0.269 µm. The standard deviation of the means was found to be 0.00134 µm, which is equal to the Type A uncertainty of the measurements, *u*_A_.

One Type B uncertainty component is the uncertainty in the magnification. As shown in the study by Lettieri and Hembree [4], the magnification uncertainty is equal to the combined standard uncertainty in the 1.0 µm SRM^®^, 0.0026 µm, multiplied times the ratio of the diameter of the 0.3 µm SRM^®^ to the 1.0 µm SRM^®^. The resulting value of *u*_m_ is equal to 0.00078 µm. The second Type B component, *u*_e_, is the uncertainty in the determination of the point in the particle image that corresponds to the actual edge of the particle. The estimated value [4] is 0.001 µm.

The combined uncertainty, obtained from the quadrature sum of *u*_A_, *u*_m_, and *u*_e_, is equal to 0.00184 µm. The effective number of degrees of freedom computed using Eq. (5) is equal to 14. In this case the coverage factor is 2.20 and the expanded uncertainty is equal to 0.0040 µm. This value is about 40 % smaller than the value currently given on the SRM^®^ 1691 Certificate.

## 4. Conclusions

- The revised expanded uncertainty (approximately 95 % confidence level) for SRM^®^ 1690 is equal to 0.005 µm with number mean diameter of 0.895 µm compared to 0.008 µm on the SRM^®^ certificates dated 2004 and earlier.
- The revised expanded uncertainty for SRM^®^ 1691 is equal to 0.004 µm with number mean diameter of 0.269 µm compared to 0.007 µm on SRM^®^ certificates dated 2004 and earlier.

## Figures and Tables

**Table 1:** Type B (systematic) Uncertainties for Measurement of SRM^®^ 1690

Type B uncertainties	Symbol	Original value, µm	Corrected value, µm
Refractive index	*u*_B1_	0.0030	0.0020
Particle doublets	*u*_B2_	0.0010	0.0010^a^
Multiple scattering	*u*_B3_	0.0010	0.0010^a^
Cell reflection	*u*_B4_	0.0010	0.0006
Finite acceptance angle	*u*_B5_	0.0005	0.0003
Optical misalignment	*u*_B6_	0.0004	0.0004^a^
$\sum_{i}\left|u_{Bi}\right|$		0.0069	
${\left[\sum_{i}{\left(u_{Bi}\right)}^{2}\right]}^{1/2}$		0.0035	0.0026

a These values are now considered to correspond to standard uncertainties.

## Footnotes

1 The symbol δ_I_, used in Mulholland et al. [1], has been replaced with *u*_Bi_.
